# Some Forms of Ethical Quackery

**Published:** 1913-08-15

**Authors:** E. O. Gillespie


### SOME FORMS OF ETHICAL QUACKERY.

BY E. O. GILLESPIE, D.D.S.

Read before the Northern Michigan Dental Society, June 5, 1913.

It is not my purpose to attempt to say any new thing. In fact, they say there is no new thing under the sun. This paper is no more than an effort to call attention to a few observations which, to me seem of sufficient importance to warrant the expenditure of a few hours time to tabulate, and a few minutes of your time to consider.

Ever since our first acquaintance with any phase of dentistry we have heard much about the Code of Ethics. The Code is sufficiently well fixed in the minds of all here as not to need review at this time. It is agreed by all that the Code is a good thing, and should every practitioner faithfully live up to the spirit of the Code the era of a Golden Day would be upon us.

We hear much of quackery in these days. In fact, there never has been a time when the profession has not been infested with a few more or less unscrupulous members. These men, with great vehemence, and some show of truth, have cried aloud that the Code is a purely arbitrary and unnecessarily tight-laced straight-jacket, which, without right or reason, and much less success, is thrust upon the professional form of every man and woman who enters our ranks. They have become bolder and bolder, and many have attained business success at least. Among these are some good and competent men. Among them have been found also some wolves in sheep's clothing—scoundrels, who care little for a good name, and less for the good name of the profession or the public whose pockets they mulct, and whose physical welfare they endanger or wreck.

I have said these men have grown bolder. Only last month one of our leading dental journals prints in full, in the most conspicuous part of the journal, an article which ridicules the Code and demands the privilege of newspaper advertising. I challenge any man to find any difference between the spirit and letter of this article and the actual performances of such concerns as "The Eureka Dental Parlors," "The Painless Dental Parlors," "The Best Dental Parlors," or other matchless masters of quackery. I think it incumbent upon all ethical dentists to send in a written protest against such publications in magazines which ask our support, and which we have a right to expect to uphold, not some abstruse, impossible or impractical set of rules, but, as I maintain, reasonable and rational rules of practice with which any honest man can easily square his professional practice.

We all know that even now the matter is being discussed by our law makers, and we are hoping that laws will be enacted whose objects will be to control the activities, to some extent at least, of this class of men. Not to force them to swallow the professional Code, for this would be about as efficacious as their professional morals, as a missionary's bible made into a dish of breakfast-food would be in satisfying a cannibal's thirst for blood; but to make it a little more difficult, for them to fool and injure the people and also to make it a little less difficult, and the task a little less thankless for such as honestly and quietly seek to serve their patients in harmony with that higher code which commands the respect and fealty of all honest men, in whatsoever field of labor.

But my purpose is not to discuss what is known to us all as plain, blunt quackery. My subject, Ethical Quackery, may sound paradoxical. With all our good intentions, and all our righteous criticism, we come back to the old truth, "A man's a man for a' that and a' that."

Not being a believer in the doctrine of Entire Sanctification, and therefore of a charitable disposition, I can easily see how many little forms of quackery unintentionally and unknowingly creep into our professional lives, even of men whose characters are above reproach, and whose lives and words are meant to be the soul of honor.

Most men are apt, under the stress of circumstances or through the demands of fashion, or unconsciously to fall into line with fads and fancies of the day and allow them to become grafted upon ourselves until they become routine practices, which, by no line of logic or rule of reason, can be separated from genuine quackery, excepting in that through frequent repetition they become wholly automatic and unintentional.

We hear much to-day, more than ever before I think, about the dignity of our profession. Dental journals are full of it. Dental associations ring with it. On the part of these writers and essayists there is a mighty effort to convince the public and a still greater effort to convince ourselves, that the dental profession is as broad, as wide, as deep, in fact as profound, as that of medicine. Allow me to quote from a recent writer: "Our profession stands to-day upon commanding ground. There is no other profession that commands such world-wide respect and honor as does American dentistry. There is a reason for this. Its vantage-ground has not been attained by half-done or slipshod work, but with a knowledge that 'whatever is worth doing at all, is worth doing well.' There was a time when medical men regarded the dentist as superfluous baggage, and that themselves gods of wit when they could allude to him as the tooth carpenter. To-day we are classed unreservedly, in the medical profession; but this tardy recognition, in view of the attitude of deferred charity in which it was given, is now resented, for the dentist is now in a position where he can hold up his head and tell the doctor to go to a place not mentioned in polite society. What the attitude of the dental toward the medical profession will be fifty years from now, the prophet will not attempt to say. However, it seems that the relative positions of these two professions are gradually reversing. I see the time in the future when the conditions that make the medical man a necessity will no longer prevail. Our present medical profession was evolved from the ancient chemists who dealt in love-charms and madstones, and searched for the elixer of youth, and the phylosopher's stone. Yes, and even drove away disease deamons with tom-toms and incantations."

I will not take my time nor yours to more than characterize this article as rubbish. I will only say the writer had much better used his time in actual, practical demonstration of the facts, or rather, statements, which called for a waste of so much time to write. Of the ego, he has a plenty, and no lamentations need be made over the loss of a part of it. This writer appears to be a mere neophite who has scarcely cast off his swaddling cloths; but this disease which I am discussing—the effort to establish our importance and dignity by loud acclaim—being about as contagious as the itch, seems to have gotten a fair hold upon him.

From time immemorial medicine, ministry, and the law have been recognized as the learned professions. Let us see with what wisdom this classification has been made. We all know with what universality it has been accepted. As for the law, these gentlemen have very often used their wits to beat, and not to better, their friend and acclaimers, the public. This will be allowed by all students of our social-political organization. All men who are in any sense students of the times and men who are keen enough to recognize the return of something resembling pure democracy as illustrated in many mighty reforms of the day, chief and foremost among which is the Commission form of government—all such men will agree that the activities of the man of the law are becoming more and more circumscribed and limited. The time may come when there will be little demand for the learned lawyer, either in chancery or in civil law, or in fact, any where else outside of criminal law.

As for the ministry, and when I say ministry I mean to cover strictly orthodox teachings of the several sects or denominations with which I am familiar, no profession on earth has so little real claim on the intelligence of man as this.

While I freely admit this falibility of the public in making a proper estimate of the relative values of the professions, I must say I believe the public's estimate of the relative importance of the medical as compared with the dental profession is much nearer the truth than that held by some of our own number. At any rate it seems to me to be very close to quackery for any of us, in view of the facts, to assume that "wiser than thou" attitude, and spend so much time in attempting to convince the people by loud claims and so little in actually producing something in the way of scientific advancement that will be conclusive.

But someone says: "We haven't time to do dentistry as demanded to-day and engage in scientific work also." That's the very kernel of the whole thing. What's true of one is true of all, practically. The very nature of our work makes it impossible for us to develop along the lines of broad knowledge incumbent upon the skilled physician and diagnostition. Limited as our field of operation is, limited as is the variety of work and conditions (I speak of pathological conditions) coming to our attention we still have enough to keep us studying and thinking, without making ourselves rediculous by persistantly, in season and out of season, forcing upon the ears of the public, and declaiming upon ourselves in such vociferous fashion that we are "something just as good." "Something just as good" always suggests that something else, presumably better, has been taken as a standard for purposes of comparison. This idle talk, then, so often heard, designed to strengthen our position, is but a palpable weakness. Even though it were wise and true, it would still be quite unnecessary for the dental profession is steadily growing in the esteem of the public; our work to-day is regarded as being of greater importance than ever before, and in the years to come it will dawn upon even the dullest mind that permanent bodily health depends very largely upon the efficiency of the chewing machine, and thus our position, about which some seem so solicitous and so fearful, must be secure as a matter of course.

But after all is said and done, we must recognize the fact that dentistry is dentistry; and while it calls for a keen, inquisitive, inventive, and progressive mind, it does not require, much less demand, that scientific study and broad knowledge so absolutely necessary in the practice of medicine in some respects.

Let us, then, admit the truth—recognize the fact that our work is largely technical and seek to become as proficient in this as possible; let us strive to educate the public as to their needs by telling them over, and over, and over again, and again, and again—without getting out of patience, and so become real teachers. This is our work, whether we wish it or not—largely the teacher and technical worker. To perpetuate the quarrel as to who is superior in service, is to demonstrate strong quackish propensities even under the shadow of the code.

Another thing which may rightly be classed as ethical quackery is the attitude of absolute subserviancy, not to say actual servility, to the public by us all. And for what I condemn in another, I would be no less severe in my own case. In my own practice I am unfortunate enough to have the field to myself. I find myself guilty very often, of this offense, but absolve myself, when making a careful introspection, with the excuse that, being alone, I must yield to public sentiment. Not to do so would, and often does bring down upon my head the charge of being too independent, because I have no competition. I am not sure this excuse will excuse.

I refer especially to doing what we are requested to do by the layman, both educated and ignorant, as to the importance of things concerning which he so glibly hands out his orders. Why, it is no uncommon thing for a patient to ask me to fill "only this one tooth in front," saying he cares nothing about the back ones; or, "I want this filled with this or that material," or, "I want a crown here or there," or "I want you to kill the nerve in this tooth, and that's all," or any one of a hundred other things which are absolutely contrary to the indications or our professional ideals.

Do you meekly submit to the dictations of hundreds of such patients during the year? Of course you do. Do I? Of course I do. But should we do so? If we are content to drift along the smooth but sickening stream of ethical quackery, *yes*. If we would place our feet upon the rugged roadway, and climb, and take our rightful place in the respect of the public, no! This thing has been so long practiced by our fellows that the public has come to look upon us as sort of hired men—men who must conform our practice to their wishes regardless of conditions—jump to their beck and call, do this or do that, as suits their fancy like some sort of "handy man" or umbrella mender. Why, it was but a week or two ago when a woman who had taken the chair and given her orders, looked at me as though she thought me either kidding or crazy when I told her I was sorry to have to inform her that her wishes could not be complied with, and that I should be pleased to consult my own knowledge of the needs of the case and do her work thus and so. She was absolutely dumbfounded and said, "Why of course you have to do it as I tell you—every other dentist I ever went to always did as I told him." What a commentary upon the dignified independence, so called, of our profession.

Only last week did I ask a prominent physician what he would do should a patient present, with a bad felon, and demand the offending finger be removed. His answer was more forceful than elegant—"Get to Hell out o' here." I do not advise the adoption of this exact language, but I do assert that the spirit is right. Imagine any selfrespecting physician submitting to the ridiculous orders from an uninformed patient that we are so often guilty of! When a man visits a physician, a lawyer, a priest, or a horse-doctor, no demands or dictations are indulged in. He knows better. It is left for us, gentlemen, of them all to do over and over again that which we know to be contrary to good practice, and may be harmful to the patient. Only when this shall cease, and our conduct in this regard warrants such respect, will the public accord that honorable and dignified position among the professions, which is now by some so eagerly sought, and by others so stoutly claimed is already accorded us. Why, then, will men continue this reprehensible form of ethical quackery? For the simple reason that it is easy to follow the line of least resistance. More men are going this way, and it is easier to go with the crowd than against it. It's a hard and rough road, this education of the public, and it takes years and it means many sacrifices; but if we *are* mechanics we are also, or should be, scientific educators. But let us beware of what sort of education we give them. It has been said that a little education is dangerous; but *I* say the *kind* of education is even more dangerous. But of that, possibly, at another time. But here lies our ethical and professional duty—to divorce ourselves, as soon as honest effort will make it possible, from this form of undignified mal-practice. I very much wish to discuss in this connection, though there may be some question as to its coming strictly under the caption of "Ethical Quackery," the fallacy of sterilization as now practiced. But I shall do no more than to raise the question at this time.

While there are several other matters which might receive attention as being inconsistent with, and needing eliminaton from, any dignified and ethical practice, and a few good things which have by the efforts of certain "dentomaniacs," been pushed out beyond the realm of reason into the field of foolish fallacy, until claims are made that are absolutely unwarranted, positions taken that are untenable, all of which render us liable to criticism. Among these subjects so abused I might mention prophylaxis, asepsis, sterilization of mouth and instruments, permanency of dental operations involving the guarantee, etc. There is one other undignified line of procedure that I will touch upon, and that briefly. And that is, advertising one's generosity by working for nothing or inadequate fees. I long for the early dawning of that day when no man shall feel the need of doing so.

The extraction of teeth free for the privilege of constructing a plate for a much less fee than the same time would command at almost any other part of our work is strictly quackery. Of this I have been guilty for years. The conditions have long been established in my community which would seem to make it compulsory, at least, until united action can be secured.

The same unnecessary practice is seen in throwing out the bait of free cleaning with every little job of $10.00 or $15.00. We all agree that of all the work in the oral cavity cleaning of the teeth is the most objectionable and often times disgusting, and withal the most important to the patient. Then what excuse is there for any of us to belittle in the mind of the patient this operation by "throwing it in" merely to represent ourselves as "good fellows?" Willing to work for nothing! Little wonder so many people look upon the prophylaxis of the mouth with so little interest and concern, if to cleanse the oral cavity is really of so little importance and value that with every little job of a few dollars, a cleaning can be thrown in. True, this free cleaning is worth just about as much as one gets for it, but this fact alone that the work of cleaning the oral cavity, the worst pest ridden area in or on the human anatomy is passed over with a "lick and a promise" is absolutely indefensible. If a set of teeth needs cleaning, let us make a professional service of it, and a thorough one, and get a respectable fee for it, regardless of what other work has been or may be done.

Men will value what they pay for, and if our patients lack in appreciation for the labor and care necessary to a good service of cleaning, the reason may not be hard to find.

